# Correlation between Physico-Chemical Characteristics of Particulated β-Lactoglobulin and Its Behavior at Air/Water and Oil/Water Interfaces

**DOI:** 10.3390/foods10061426

**Published:** 2021-06-19

**Authors:** Franziska Kurz, Vera Reitberger, Claudia Hengst, Christine Bilke-Krause, Ulrich Kulozik, Jannika Dombrowski

**Affiliations:** 1 Chair of Food and Bioprocess Engineering, TUM School of Life Sciences, Technical University of Munich, Weihenstephaner Berg 1, 85354 Freising, Germany; vera.reitberger@mytum.de (V.R.); claudia.hengst@tum.de (C.H.); ulrich.kulozik@tum.de (U.K.); Jannika.Dombrowski@rd.nestle.com (J.D.); 2 Krüss GmbH, Borsteler Chaussee 85, 22453 Hamburg, Germany; c.bilke-krause@kruss.de; 3 Nestlé Research, Société des Produits Nestlé SA, Route du Jorat 57, 1000 Lausanne 26, Switzerland

**Keywords:** β-lactoglobulin, thermal aggregation, particle size, zeta potential, cross-linking degree, interfacial properties, foam, emulsion

## Abstract

It is widely accepted that protein-based particles can efficiently stabilize foams and emulsions. However, it is not fully elucidated which particle properties are decisive for the stabilization of air/water and oil/water interfaces. To unravel this correlation, selected properties of nano-sized soluble β-lactoglobulin particles were changed one at a time. Therefore, particles of (1) variable size but similar zeta potential and degree of cross-linking and (2) similar size but different further properties were produced by heat treatment under a specific combination of pH value and NaCl concentration and then analyzed for their interfacial behavior as well as foaming and emulsifying properties. On the one hand, it was found that the initial phase of protein adsorption at both the air/water and the oil/water interface was mainly influenced by the zeta potential, independent of the particle size. On the other hand, foam stability as resolved from the time-dependent evolution of mean bubble area negatively correlated with disulfide cross-linking, whereas emulsion stability in terms of oil droplet flocculation showed a positive correlation with disulfide cross-linking. In addition, flocculation was more pronounced for larger particles. Concluding from this, foam and emulsion stability are not linked to the same particle properties and, thus, explanatory approaches cannot be used interchangeably.

## 1. Introduction

Protein particles have attracted considerable attention during the last few years, particularly in the context of stabilizing foams and emulsions as they are deemed to provide high product stability and offer novel opportunities for the food industry [1,2,3]. However, based on the current state of the literature, the correlation between specific particle properties and high foam as well as emulsion stability has still not been fully elucidated, i.e., knowledge of decisive particle properties for different applications as well as interface types is lacking. In this regard, a mechanistic understanding of how those particles act at both air/water and oil/water interfaces is crucial for their design and applicability. Due to the deformable and porous structure of protein particles, a transfer of the classical contact angle-based ‘Pickering concept’ is not considered reasonable [4,5,6]. Thus, to better understand and explain their functionality in contrast to inorganic particles or to proteins in their native state, in depth knowledge about their behavior at air/water and oil/water interfaces is required.

According to the literature, several physicochemical characteristics are referred to as being important for the formation and stabilization of protein-based foams and emulsions. These include the proteins’ particle size, degree of (intramolecular) cross-linking, surface charge (zeta potential), and surface hydrophobicity [1,2,3,7]. However, there is no consensus on their prioritization as well as specific relevance for protein particle-based foams or emulsions. 

Whey proteins have often been used as a model system to produce (heat-induced) protein-based particles and to evaluate their behavior at air/water or oil/water interfaces, as described in detail in the following.

Concluding from a multitude of foam-related studies, it is generally recognized that protein diffusion to the interface and thus foamability decreases with increasing particle size [8,9,10]. However, the lowering of the protein particles’ surface charge (zeta potential) via pH adjustment or salt-mediated charge screening leading to a reduced electrostatic barrier of adsorption as well as an increase in surface hydrophobicity seems to be even more important in view of foamability than particle size [11,12,13,14]. By contrast, foam stability seems to positively correlate with particle size and negatively correlate with zeta potential [8,12,13]. By contrast, [15] reported contradicting findings regarding the influence of the zeta potential. Moreover, the degree of cross-linking (i.e., the structural flexibility) is known to influence foam stability [10,12,16]. Regarding the degree of cross-linking, however, the drawing of generalized conclusions is quite difficult. This is due to the variety of cross-linking techniques used in different studies such as heat-induced or enzymatic cross-linking as well as the lack of a common definition of the ‘degree of cross-linking’ [12,14,16,17]. 

In terms of emulsions or oil/water interfaces, similar factors are described to affect their formation and stabilization [18,19,20,21,22,23,24]. However, in contrast to foams, oil droplet flocculation and thus emulsion stability was found to be negatively affected by the screening of the particles’ zeta potential [18,22]. Surface hydrophobicity seems to be decisive in terms of the emulsifying activity; however, the effect was less pronounced compared to its impact on foamability [19]. 

Overall, this shows that the interrelation and priority of the different particle physicochemical properties in terms of foam and emulsion formation as well as stabilization are still not fully elucidated. This can be explained as follows: First, many studies varied more than one particle characteristic at a time (e.g., particle size and zeta potential or particle size and surface hydrophobicity), wherefore it is difficult to assess their respective specificity. Second, most studies focused on either air/water or oil/water interfaces and used different particle formation as well as characterization methods. 

Therefore, to approach this knowledge gap, the objective of this work was (i) the controlled production of protein-based particles with one physicochemical property changed at a time to (ii) unravel the correlation between particle characteristics and their behavior at the air/water and the oil/water interface. For this, the main whey protein β-lactoglobulin (β-Lg) was used because its structure is well-characterized and its aggregation behavior can be influenced in a targeted manner [25,26,27]. In detail, particles of (1) variable size but similar zeta potential and degree of cross-linking and (2) similar size but different further physicochemical properties were produced by heat treatment of β-Lg solutions (i.e., c = 10 g L^−1^, 80 °C/90 min) at different pH values (i.e., 5.8 to 8.5) and in the presence of NaCl at various molarities (i.e., 0–60 mM). The obtained β-Lg particles were then analyzed for their interfacial behavior as well as foaming and emulsifying properties. Particle purity in terms of the absence of remaining native β-Lg molecules and NaCl was achieved by an isoelectric precipitation in combination with a centrifugal separation and subsequent re-dissolution of the particle pellet.

Following this, conclusions regarding a correlation between specific physicochemical particle properties and the surface/interfacial behavior as well as foam/emulsion stabilization capability shall be drawn. Moreover, this was supposed to provide some general conclusions guiding the practical optimization of the stability of foams and emulsions stabilized by protein-based particles.

## 2. Materials and Methods

### 2.1. Materials

Bovine β-lactoglobulin (β-Lg) (A and B) was purified from whey protein isolate (WPI 895, Fonterra Co-operative Group, Auckland, New Zealand) by selective thermal aggregation with subsequent microfiltration/ultrafiltration and spray drying, as described in detail by [28]. The obtained powder had a total protein content of >99% on a dry matter basis (Vario MAX cube, Elementar Analysensysteme GmbH, Langenselbold, Germany; RP-HPLC), whereby >99% was β-Lg. Native β-Lg represented >86% of the total protein as determined by RP-HPLC (Toro-Sierra et al. 2013). Lactose (HPLC) and salts (Na, K, Ca) (ELEX 6361, Eppendorf AG, Hamburg, Germany) concentrations were <0.05% and 0.7%, respectively.

### 2.2. Production of β-Lactoglobulin Particles by Thermal Treatment and Particle Concentration

β-Lg powder was dissolved in deionized water (Millipore Corporation, Bedford, MA, USA) or in NaCl solutions of different molarities (20 or 60 mM) (Merck KGaA, Darmstadt, Germany) to a total protein content of 10 g L^−1^. For the determination of protein content during particle production and purification, a method according to Dumas was used (Elementar Analysensysteme GmbH, Hanau, Germany). The solutions were stirred for 12 h at 4 °C to ensure complete hydration.

*Production of β-Lactoglobulin Particles by Thermal Treatment*

Prior to heat treatment, the solutions were tempered to 20 °C and the pH was adjusted to 5.8, 6.2, 6.8, 7.6 or 8.5 with 1 M HCl (Merck KGaA, Darmstadt, Germany) or 1 M NaOH (Merck KGaA, Darmstadt, Germany). The formation of β-Lg particles was conducted at a temperature of 80 °C for a heat holding time of 90 min using a thermostatically controlled water bath. The solutions were stirred during heating and their temperature was monitored by a sensor connected to an Almemo 2590-4AS (Ahlborn Mess-und Regelungstechnik, Holzkirchen, Germany). The denaturation was stopped by cooling the solutions to 20 °C in ice water. 

*Determination of the Concentration of Particles after Heat Treatment*

Due to the dependence of β-Lg´s denaturation reaction rate constant on the pH value and salt concentration during heating, a native fraction of β-Lg molecules could remain in the particle solution after heat treatment. In order to determine the residual native β-Lg, the denatured proteins (i.e., the protein particles) were precipitated at pH 4.6 as described by [28]. The native and the denatured proteins were separated by using a syringe filter (pore size 0.45 µm, Chromafil Xtra RC-45/25, Macherey-Nagel GmbH & Co. KG, Düren, Germany). The protein content of the native fraction, i.e., the filtrate, was determined by reversed phase high performance liquid chromatography (RP-HPLC, Agilent 1100 Series Chromatograph, Agilent Technologies Inc., Santa Clara, CA, USA) as reported by [29]. For this, 200 μL of the solution was dissolved in 800 μL guanidine buffer and left for 30 min at room temperature to ensure complete solubilization (i.e., denaturation and cleavage of disulfide bonds) of the protein molecules [29]. The total protein content, native and denatured, was determined accordingly; however, no precipitation step at pH 4.6 was conducted.

The degree of denaturation (*DD*) was calculated by the following equation (Equation (1)):

(1) $$DD\;\left(\%\right)=\left(1-\frac{C_{t}}{C_{0}}\right)\cdot 100\%$$

*C*_*t*_ is the native β-Lg concentration in a pH 4.6 precipitated sample, whereas *C*_0_ is the native β-Lg concentration in the unheated solution.

*Particle Purification*

In order to exclude the influence of remaining native β-Lg molecules as well as the NaCl on the interfacial investigations, a purification step was necessary, in some cases, to obtain a purified (*DD* ≥ 96%, conductivity (κ) < 1 mS cm^−1^) particle solution. Therefore, a concentration–purification step based on an isoelectric precipitation was performed after heat treatment in order to reduce the content of remaining native protein and NaCl [30]. The pH of the heated protein solution was adjusted to 4.6. Consequently, the denatured protein particles precipitated, whereas the native proteins as well as the salt remained soluble. To accelerate the separation process, the samples were centrifuged at 500× *g* for 15 min at 20 °C (38.5 g sample solution per centrifuge tube) using the Multifuge 1S-R (Thermo Fisher Scientific, Waltham, MA, USA). The supernatant was discarded and the particle pellet was re-dissolved in deionized water. The volume of deionized water was chosen to achieve a total protein concentration of about 1.0% (Elementar Analysensysteme GmbH, Hanau, Germany). In addition, 1 M NaOH was added to adjust the pH to 6.8. After at least 2 h (~12 h: pH 5.8/0 mM; pH 6.2/0 mM; and pH 8.5/60 mM) of gentle shaking, the concentration–purification step was repeated until a *DD* of ≥ 96% (monitored by RP-HPLC) and a conductivity (κ) < 1 mS cm^−1^ (S47—SevenMulti™, Mettler-Toledo AG, Schwerzenbach, Switzerland) was achieved. Subsequently, the total protein concentration of the purified particles (i.e., pH 6.8, *DD* of ≥ 96%, κ < 1 mS cm^−1^) was adjusted to 8.2 g L^−1^ by using deionized water. 

All experiments were carried out at a temperature of 20 °C using a protein concentration of 8.2 g L^−1^ (determined by RP-HPLC) and a pH of 6.8. Apart from that, the solutions with particles produced at pH 5.8 (0 mM) and at pH 6.2 (0 mM) were additionally investigated at pH values of 6.2 and 6.3, respectively.

### 2.3. Quantification of Protein Thiols and Disulfide Bonds

In order to determine the concentration of thiols, which are part of disulfide bonds, the concentration of free and total (free thiols and thiols associated in disulfide bonds) thiols was quantified by RP-HPLC using the thiol reagent 4,4′-Dithiodipyridine (DTDP) as described by [31]. In brief, free thiols (and reduced disulfide bonds) react with DTDP forming 4-Thiopyridine (4-TP), which absorbs at 324 nm in the pH range from 3–7. Therefore, the concentration of thiols associated in disulfide bonds c_RSSR_ can be calculated by the difference between the concentration of free thiols and total thiols. In order to determine the degree of disulfide cross-linking and thus the structural flexibility, the change in the concentration of disulfide bonds due to processing was used according to the following equation (Equation (2)):

(2) $$\;\;\;\;\;\;\;\;\;\;\;\;degree\;of\;disulfide\;cross-linking\;\left(\%\right)=\frac{c_{RSSR\;\beta -Lg\;aggregates}}{c_{RSSR\;untreated\;\beta -Lg}}\cdot 100\%$$

### 2.4. Particle Size and Zeta Potential Measurements

The volume-based particle size distributions as well as the median volume-based particle size (d_50.3_) were measured by dynamic light scattering using a Zetasizer Nano ZS (Malvern Panalytical GmbH, Kassel, Germany). Samples were measured in 173° backscatter mode 10 times for 1 min after an equilibration time of 2 min. The refractive index (RI) used for calculation was set to 1.344. Results of the particle size measurements are means of two samples of at least two individual experiments. 

The zeta potential of the unheated and purified protein solutions was measured by a Zetasizer Nano ZS System (Malvern Panalytical GmbH, Kassel, Germany). The measurement consisted of two runs. The obtained electrophoretic mobility was converted to the zeta potential by the Zetasizer Software 7.13 (Malvern Panalytical GmbH, Kassel, Germany). Results of the zeta potential measurements are means of at least two individual experiments with three samples each.

### 2.5. Viscosity Measurements

Viscosity of the purified solutions was determined on the Modular Compact Rheometer MCR 302 (Anton Paar GmbH, Graz, Austria) using a double gap geometry (DG 26.7, Anton Paar GmbH, Graz, Austria). The used procedure included an increasing shear rate ramp from 0.1 to 100 s^−1^ followed by a decreasing ramp from 100 to 0.1 s^−1^. For data analysis and calculation of the dynamic viscosity, the software RheoPlus and RheoCompass 1.22 were used. Thereby, all samples showed Newtonian behavior. Results of the measurements are means of at least two individual experiments.

### 2.6. Surface and Interfacial Tension

The surface tension (*σ*) at the air/water interface as well as the interfacial tension (*γ*) at the oil/water interface were measured by the pendant drop method using the Drop Shape Analyzer DSA 100 (Krüss GmbH, Hamburg, Germany) equipped with a pendant drop module (Krüss GmbH, Hamburg, Germany). A drop containing the purified particle solutions was formed automatically at the tip of a cannula of a syringe into either air or a cuvette containing 100% caprylic acid (Primal State Performance GmbH, Berlin, Germany). Caprylic acid was used due to its negligible surface activity compared to vegetable oil (data not shown). The change in the bubble radius and shape was recorded for a time period of 3600 s. The surface tension and the interfacial tension were calculated by the Young-Laplace-Fitting using the Advance software (Krüss GmbH, Hamburg, Germany). Therefore, the needle diameter and the densities of air or oil and the water phase were considered for the calculation. Results of the surface/interfacial measurements are means of at least two individual experiments. In order to better compare the interfacial properties of the specific particles at the air/water and oil/water interface, the surface pressure (*π_σ_*(*t*)) and the interfacial pressure (*π_γ_*(*t*)) were calculated additionally as follows (Equations (3) and (4), respectively):

(3) $$\pi_{\sigma }\left(t\right)\left(mN\;m^{-1}\right)=\sigma_{0}-\sigma \left(t\right)$$

(4) $$\pi_{\gamma }\left(t\right)\left(mN\;m^{-1}\right)=\gamma_{0}-\gamma \left(t\right)$$

Here, *σ_0_* (*γ_0_*) is the surface tension (interfacial tension) of deionized water and *σ*(*t*) (*γ*(*t*)) is the time-dependent surface tension (interfacial tension) of the β-Lg particle sample solutions. 

### 2.7. Foaming and Characterization of Foam Stability

Foams were prepared by air sparging (foam height = 180 mm, $\dot{V}$ = 0.2 L) through a porous frit (pore size 9–16 µm) into the β-Lg particle solution (V = 50 mL, c = 8.2 g L^−1^) by using the Dynamic Foam Analyzer DFA100 (Krüss GmbH, Hamburg, Germany). The pH values of the respective β-Lg solutions were kept at 6.8. Apart from that, the solution with particles produced at pH 5.8 (0 mM) was additionally investigated at a pH value of 6.2. The evolution of the bubble area distribution was recorded over a time period of 3600 s by the attached Foam Structure Module (DFA100FSM, Krüss GmbH, Hamburg, Germany). The camera was thereby located at a column height of 65 mm. Thereof, the mean bubble area was determined by using the software Advance (Krüss GmbH, Hamburg, Germany). The obtained mean bubble area (A) in dependence of time (t) was used to describe and compare the structural decay behavior of the different foams. According to [12], the number of air bubbles within a foam can be characterized by an exponential foam decay, revealing an increase in the air bubble area during coarsening. The plotting of the air bubble area against time thereby allows for the estimation of the coarsening exponent α. Therefore, a power-law fit of the experimental data according to A ~ t^α^ was performed (t_>50 s_ > air sparging for foam formation) to evaluate the foam bubble stabilization behavior. 

Results of the foaming experiments are means of at least two individual experiments. Details about the measuring principle are described by [32]. 

### 2.8. Emulsification and Measurement of Oil Droplet Size

Oil-in-water emulsions (10/90 (*w/w*)) with sunflower oil (THOMY, Nestlé Deutschland AG, Neuss, Germany) and β-Lg particles (c = 8.2 g L^−1^) in the aqueous phase were produced by high pressure homogenization (APV 1000, SPX Corporation, Inc., Soborg, Denmark) at 300 bar. The pH values of the respective β-Lg solutions were kept at 6.8 prior to adding the oil. Apart from that, the solution with particles produced at pH 5.8 (0 mM) was additionally investigated at a pH value of 6.2. A pre-mix was produced by diluting the oil in the protein–water phase under stirring at 300 rpm followed by stirring at 6000 rpm for 60 s using an ultra turrax (ULTRA-TURRAX^®^ T 25 digital (S25N), IKA^®^-Werke GmbH & Co. KG, Staufen, Germany). Thereof, the measurements of the particle size distribution and the median volume-based particle size (d_50.3_) as well as the surface weighted mean (d_3.2_) of the freshly prepared emulsions were carried out with the Mastersizer 2000 with a Hydro 2000 unit (Malvern Panalytical GmbH, Kassel, Germany). In order to determine the emulsion stability, the flocculation factor (*ff*) was used as calculated according to the following equation (Equation (5)):

(5) $$flocculation\;factor\;\left(-\right)=\frac{d_{50.3\;\left(without\;SDS\right)}}{d_{50.3\;\left(with\;SDS\right)}}$$

Therefore, 1 mL of the emulsion sample was mixed with 9 mL of 0.5% sodium dodecyl sulfate (SDS) solution and measured as described before. Results of the oil droplet measurements are means of at least two individual experiments.

## 3. Results and Discussion

### 3.1. Preparation and Structural Characterization of β-Lg Particles

The variation of the pH value and the NaCl concentration during heat treatment of the β-Lg solutions allowed for the formation of soluble (not sedimenting) particles of different sizes and degrees of denaturation (i.e., remaining native protein after heat treatment), as can be seen in Table 1. 

**Figure 1:**
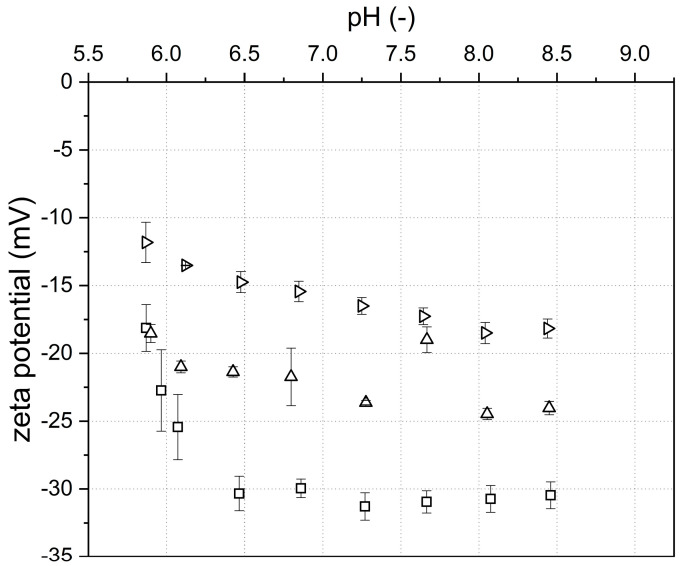
Zeta potential of native β-Lg solutions (c = 10 g L^−1^) as a function of the pH at varying NaCl concentrations (□ 0, △ 20, and ▷ 60 mM) before heat treatment. Error bars indicate standard deviation.

Thereby, all samples had monodisperse size distributions except for two pH–NaCl combinations (i.e., pH 5.8/60 mM NaCl and pH 6.2/60 mM NaCl), which showed a polydisperse distribution (data not shown). Generally, particle size increased with decreasing pH. This can be attributed to the reduced range of electrostatic interactions between protein molecules upon approaching the isoelectric point (IEP) of the native β-Lg (d_50.3_ 4 nm), which is around 5.1 [8,33]. In consequence, hydrophobic interactions become more dominant, leading to an increased aggregation tendency. In addition, the presence of ions leads to less pronounced repulsive forces and in consequence to an increased aggregation probability of β-Lg. Thereby, the effective range of electrostatic forces decreases with increasing concentration of ions (Figure 1). Therefore, increasing the concentration of NaCl at constant pH caused a slight increase in particle size.

**Figure A1:**
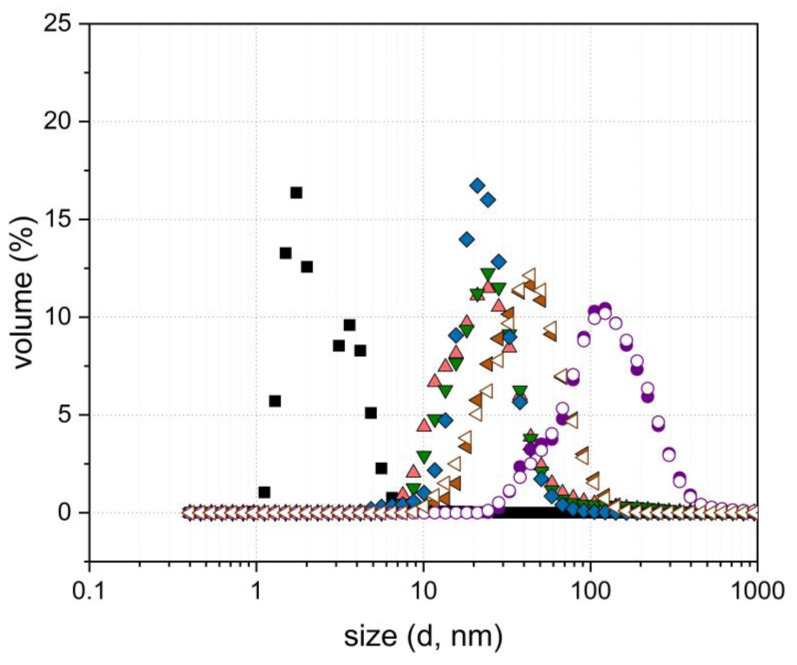
Particle size distribution curves of β-Lg particles (c = 8.2 g L^−1^, pH 6.8) after purification (heating conditions (pH/NaCl): ■ pH 8.5/0 mM, ◆ pH 6.8/0 mM, ◀ pH 6.2/0 mM, ● pH 5.8/0 mM, ▼ pH 6.8/20 mM, and ▲ pH 8.5/60 mM). Non-filled symbols indicate an additional variation in the pH value (i.e., pH ≠ 6.8) after purification (◁ pH 6.2/0 mM (pH 6.3) and ○ 5.8/0 mM (pH 6.2)).

Besides the particle size, the reaction rate constant *k* for particle formation also depends on the pH value. As a result, the concentration of the remaining native protein after heat treatment varies [34,35]. Heat treatment of the different β-Lg solutions therefore did not always result in a full denaturation, as can be seen from the values of the degree of denaturation (*DD*), which varied from 69 to >96% (Table 1). Thus, in order to exclude the influence of remaining native protein as well as NaCl on interfacial and foaming/emulsifying properties, the remaining native molecules in the heat-treated samples were removed by purification steps until the degree of denaturation *DD* and the electric conductivity κ reached ≥ 96% and < 1 mS cm^−1^, respectively. Thereby, particles of different size clusters could be produced (Table 2), which allowed for the investigation of particle size-related techno-functional properties (i.e., foaming and emulsification). It is important to note that resuspending the pellets of samples heat-treated at pH 6.2, 6.8, and 7.6 in the presence of 60 mM NaCl did not result in the formation of separated particles after purification. This may be due to incomplete resolubilization based on reinforced attractive interactions. Thus, the purification method using centrifugation and resuspension was not suitable to process these particles. These samples were therefore excluded from further investigations. The size of the other particles was not changed markedly by purification, with the exception of the ones produced at pH 5.8. The size distributions of the purified particles are shown in Appendix A (Figure A1).

As summarized in Table 2, the purified particles were also analyzed for their zeta potential as well as their disulfide cross-linking degree. An increase in the degree of cross-linking corresponded to an increase in disulfide cross-links (i.e., a decrease in the particle structural flexibility). It can be deduced that particles of a similar size range (~22 nm) (pH 6.8/0 mM NaCl, pH 6.8/20 mM NaCl, and pH 8.5/60 mM NaCl) and zeta potential (~−30 mV) but different degrees of disulfide cross-linking were produced.

**Figure A2:**
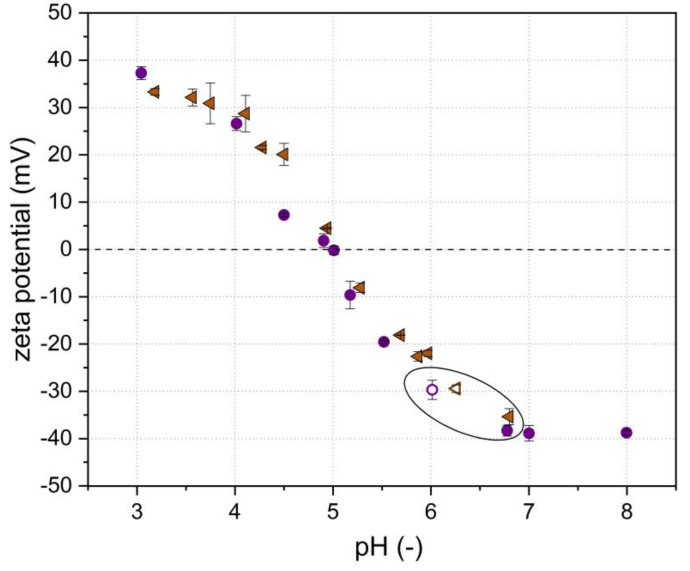
Zeta potential of purified β-Lg particles (c = 8.2 g L^−1^) obtained at different heating pH values (● pH 5.8, ◀ pH 6.2) as a function of the pH. Error bars indicate standard deviation.

In addition, the pH value of the particles with a given median diameter of 44 and 120 nm was changed to modify the zeta potential. Due to this, the investigation of the influence of different particle sizes (3, ~22, ~42, ~120 nm) independent of the zeta potential (~−30 mV) was possible. Therefore, the zeta potential of these particles was adjusted to ~−30 mV corresponding to pH 6.2 and 6.3, respectively (Figure A2). Thereby, no significant effect of pH adjustment on the particle size distributions was observed (Table 2). Furthermore, it can be assumed that the degree of cross-linking does not change due to the change in pH value after heat treatment, since, e.g., a certain temperature increase is necessary to induce the reactivity of the free thiol group [36].

In addition, the dynamic viscosity of the samples ranged between 1.10 (heating pH 5.8) and 1.39 mPas (heating pH 8.5), whereby the differences were not significant. This allows for the exclusion of viscosity effects with regard to both interfacial adsorption and foam destabilization (e.g., foam drainage).

To sum up, the applied method allowed for the production of purified β-Lg particles with variable physicochemical properties (i.e., size, zeta potential, and degree of disulfide cross-linking), as summarized in Table 2. Therewith, the specific relevance of these properties for the particles‘ interfacial properties, considering both the air/water and the oil/water interface, could be assessed.

### 3.2. β-Lg Particles at the Air/Water Interface: Interfacial Properties and Foam Structure Dynamics

#### 3.2.1. Interfacial Properties

**Figure 2:**
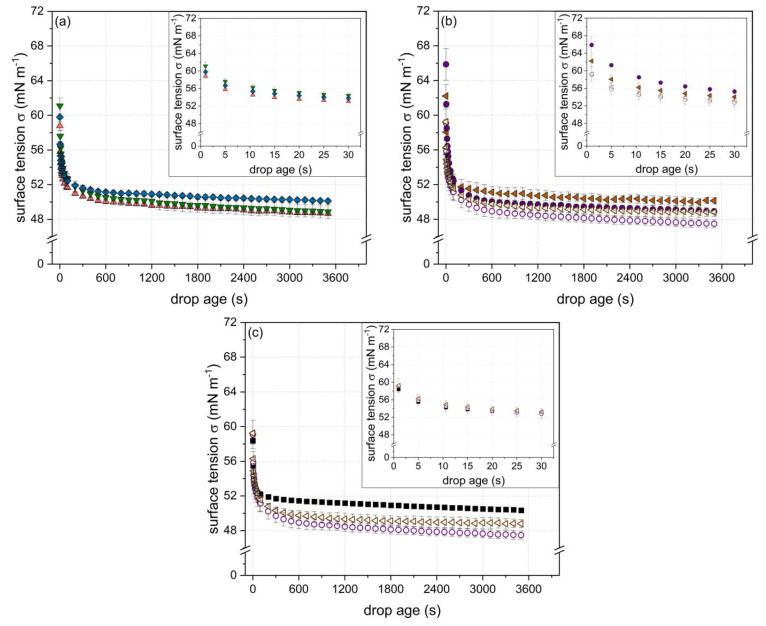
Evolution of surface tension (σ) in dependence of time as a function of the β-Lg particle (c = 8.2 g L^−1^) physicochemical properties: (**a**) varying degree of cross-linking (▼ 85, ▲ 92, and ◆ 109%) but similar size (~22 nm) and similar zeta potential (~−30 mV); (**b**) varying net zeta potential (● −38 and ○ −30 mV or ◀ −35 and ◁ −29 mV, respectively) but similar size (●/○ ~120 and ◀/◁ ~42 nm, respectively) and similar degree of cross-linking (~105%); (**c**) varying particle size (■ 3, ◁ 40, and ○ 119 nm) and degree of cross-linking (■ 120 and ◁/○ ~105%) but a similar zeta potential of ~−30 mV. Error bars indicate standard deviation.

In order to investigate the interfacial behavior of specific particle physicochemical properties (i.e., particle size, zeta potential, and degree of cross-linking (Table 2)), the evolution of surface tension in dependence of the particles’ physiochemical characteristics during a period of 3600 s was determined. In general, all particles reduced the surface tension from 72 to around 50 mN m^−1^, as can be seen in Figure 2a–c. The respective insets in Figure 2a–c highlight the initial decrease in the surface tension.

(a) Surface tension as a function of degree of cross-linking

Figure 2a shows the evolution of the surface tension for particles of similar size (~22 nm) and zeta potential (~−30 mV) but different degrees of disulfide cross-linking (85, 92, and 109%). According to the literature, the cross-linking degree, i.e., the molecular flexibility, is important in terms of rearrangement at the interface. Thus, it plays a role at longer time scales rather than in the initial phase of adsorption. Similar conclusions were drawn by [10] using enzymatic cross-linked α-lactalbumin particles. Hence, as expected, no specific effect of the degree of cross-linking during the initial phase (*t* < 5 s) of adsorption was observed. For longer time scales, it appeared that dependent on the cross-linking degree, the surface tension reduction varied among the samples. Thereby, the decrease in surface tension was found to be more pronounced for particles with a lower (<100%) cross-linking degree. Due to the greater reduction in surface tension of the less cross-linked particles subsequent to the initial phase, the curve corresponding to the particles of the lowest cross-linking degree and the curve corresponding to the particles of the highest cross-linking degree crossed each other (in the time range of 20 ≤ *t* < 2000 s). The effect became most evident for *t* > 2000 s; however, it was not significant (*p* > 0.05; one way ANOVA analysis with Tukey post hoc test performed by Origin Lab 2018). Nonetheless, the results shown in Figure 2a indicate a more pronounced rearrangement of less-cross-linked particles at the air/water interface.

(b) Surface tension as a function of zeta potential and particle size

Figure 2b shows the surface tension as a function of time for particles of two size classes (~42 and ~120 nm) with different zeta potentials, respectively (~42 nm: −35 and −30 mV; ~120 nm: −38 and −30 mV) (Table 2). The degree of cross-linking of the particles was similar (~105%). Comparing the particles of similar size but different zeta potential (filled and non-filled symbols), a stronger initial decrease in surface tension (i.e., in the range of *t* < 5 s) can be detected with decreasing zeta potential. As it has been shown for proteins in general, an energetic barrier must be overcome to allow for adsorption at the air/water interface [11,37]. This adsorption barrier can be reduced by decreasing the zeta potential of a particle, resulting in a higher adsorption speed [11,33,38].

Comparing the particles of different size (~42 and ~120 nm) but similar zeta potential (−30 mV) (non-filled symbols), no significant difference in their potential to reduce surface tension could be detected (*t* < 3000 s). A decrease in particle size is in general associated with a stronger decrease in the initial surface tension [9,10,12]. However, according to the obtained results, the particle size was clearly less important than the zeta potential in terms of adsorption at the interface. However, particle size could become more evident as an influential factor at greater differences in size and lower particle concentrations.

Interestingly, towards longer time scales (*t* > 100 s), the rate of surface tension reduction was higher for the larger particles (~120 nm) than for the smaller ones (~42 nm), despite the similarities in zeta potential (~−30 mV) and cross-linking degree (~105%). The same trend could be detected at higher net zeta potentials. It has to be noted that although particles exhibited a similar degree of cross-linking (Table 2), they might have differed in their disulfide bond distribution. Due to the influence of the pH value and the heating conditions (temperature and time) on the reactivity of the free thiol group and, thus, the formation of disulfide cross-links [35], inhomogeneous distributions of disulfide bonds within the particle can occur. This aspect in turn can affect the rearrangement of the particles after adsorption at the interface [39,40]. Thereof, the detected differences in the surface tension reduction at longer time scales are likely to be due to the differences in the distribution of the disulfide cross-links within the particles limiting the interfacial rearrangement based on the distribution of the cross-links.

(c) Surface tension as a function of particle size and degree of cross-linking

In order to unravel the effect of the particle size itself, Figure 2c shows the time-dependent evolution of the surface tension for particles differing in size (3, 40, and 119 nm) but having a similar zeta potential of ~−30 mV (Table 2). In accordance with the results shown before, no significant influence of the particle size on the initial surface tension reduction could be detected despite the greater magnitudes of size. Thus, the decrease in zeta potential appeared to balance out the decelerated diffusion rate related to an increasing particle size in the investigated particle size range.

However, at longer time scales (*t* > 100 s), the smallest particles exhibited higher surface tension values compared to the larger ones. This can be related to the higher degree of cross-linking of the smallest particles (120%) compared to the larger ones (~105%) (Table 2), and thus, to a less effective rearrangement at the air/water interface as stated above.

#### 3.2.2. Foam Stability

**Figure 3:**
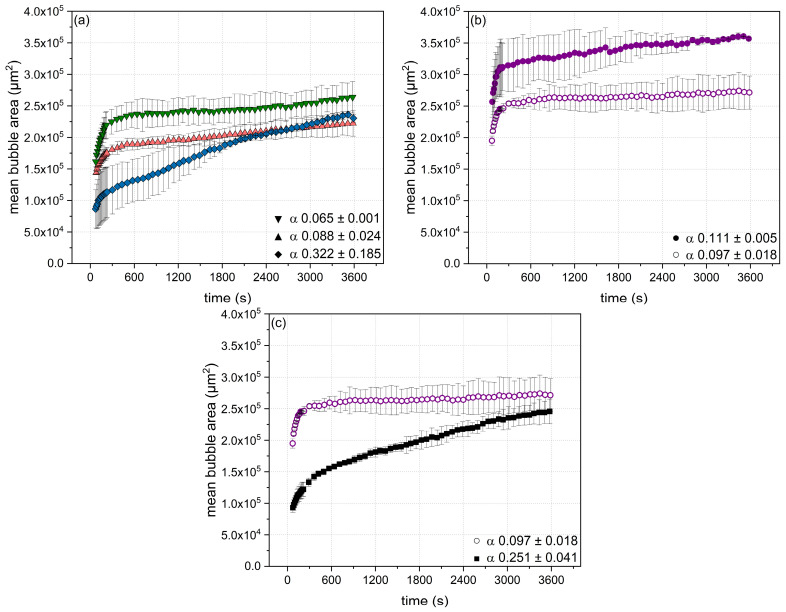
Evolution of the mean bubble area in dependence of time as a function of the β-Lg particle (c = 8.2 g L^−1^) physicochemical properties: (**a**) varying degree of cross-linking (▼ 85, ▲ 92, and ◆ 109%) but similar size (~22 nm) and similar zeta potential (~−30 mV); (**b**) varying zeta potential (● −38 and ○ −30 mV) but similar size (~120 nm) and similar degree of cross-linking (104%); (**c**) varying size (■ 3 and ○ 119 nm) and degree of cross-linking (■ 120 and ○ 104%) but similar zeta potential (~−30 mV) including the coarsening factor α. Error bars indicate standard deviation.

In order to investigate the influence of specific particle physicochemical properties (i.e., particle size, zeta potential, and degree of cross-linking (Table 2)) decoupled from each other on air bubble stability, the mean bubble area (A) as a function of time (t) was analyzed. The obtained data were used to calculate the coarsening factor (α) according to A ~ t^α^ as described by [12]. The results are displayed in Figure 3a–c.

(a) Mean bubble area as a function of degree of cross-linking

Figure 3a shows the time-dependent evolution of the mean bubble area for β-Lg particles of similar size (~22 nm) and similar zeta potential (~−30 mV) but variable degrees of disulfide cross-linking (Table 2). As can be seen, the mean bubble area rapidly increased within the first 500 s, whereby the foams formed by particles with the highest degree of cross-linking (109%) exhibited the smallest mean bubble area. However, for measurement times longer than 500 s, the mean bubble area of foams from particles with a high degree of cross-linking (109%) continued to increase more strongly (almost linear) as compared to foams stabilized by particles having a lower degree of cross-linking (<100%). As a result, bubble coalescence was mitigated by decreasing the particles’ cross-linking degree as reflected in the decreasing coarsening factor (α) values. In turn, this means that those foams had higher stability. Potentially, this increase in stability could be due to network formation of the less cross-linked particles within the interfacial film subsequent to the adsorption and rearrangement at the interface, resulting in an increase in the elasticity of the formed surface film and, thereby, a higher resistance against destabilization [16,41]. Besides, a lower cross-linking degree is assumed to promote the formation of regular and, thus, high packed interfacial layers at high particle concentrations at the interface or at late adsorption stages, thereby increasing the foam stability [41,42].

(b) Mean bubble area as a function of zeta potential

When comparing the foaming behavior of particles of similar size (~120 nm) and disulfide cross-linking (104%) but different zeta potential (−30 and –38 mV), it appeared that a decrease in zeta potential resulted in the formation of smaller air bubbles after foaming (<100 s) (Figure 3b). This can be explained by the faster initial surface tension decrease (Figure 2b) due to reducing the electrostatic barrier of adsorption [37]. The evolution of the mean bubble area in dependence of time generally showed a high bubble stability, as can be seen by the rather constant mean bubble area up to 3600 s. However, the effect was slightly more pronounced for the particles exhibiting a lower zeta potential.

(c) Mean bubble area as a function of particle size and degree of cross-linking

As can be seen in Figure 3c, particle size had a clear effect on the initial mean bubble area and its evolution over time. At a similar zeta potential of ~−30 mV, the smallest particles (3 nm) resulted in the formation of air bubbles of half the area of those from the largest particles (119 nm) directly after foaming. However, beginning at about 250 s after foam formation, the larger particles kept the bubble area rather constant (α: 0.097), whereas the mean bubble area of foams from smaller particles continued to increase (α: 0.251). Thus, the smaller ones were able to occupy the air/water interface faster, but they were not able to lastingly stabilize the bubbles. The enhanced foam stability in the presence of larger particles could be due to a blocking of the foam lamellae by non-adsorbed particles resulting in a decreased drainage and, thus, keeping the bubbles apart from each other [13,15]. In addition, a lower cross-linking degree of the larger particles (119 nm) compared to the smaller ones (3 nm) allowed for a better rearrangement at the air/water interface and, thus, the formation of more cohesive films based on more pronounced particle–particle interaction imparting higher foam stability [8].

**Figure 4:**
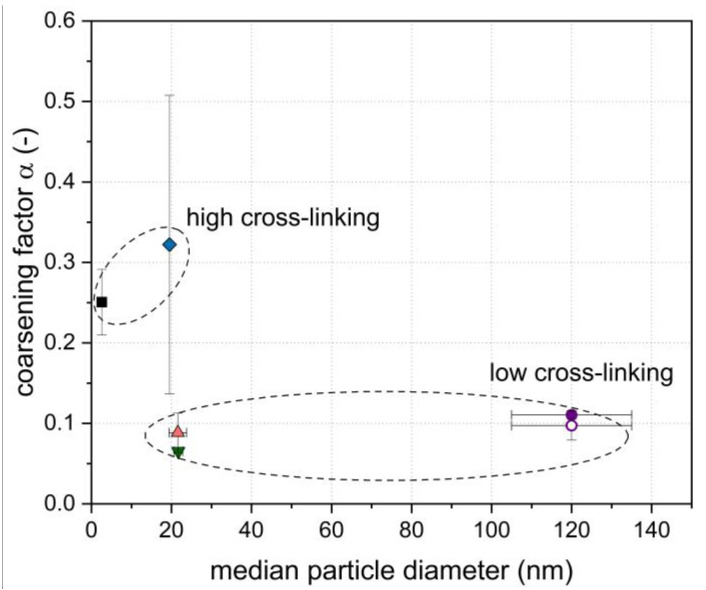
Coarsening factor α in dependence of the median particle size as a function of the cross-linking degree (▼ 85, ▲ 92, ●/○ 104, ◆ 109, and ■ 120%; c_particles_: 8.2 g L^−1^). Error bars indicate standard deviation.

As can be seen in Figure 4, an increase in the coarsening factor α (i.e., a lower bubble stability) was found to correlate with an increase in the cross-linking degree, whereby a variation in the particle size only showed minor effects. In contrast, the zeta potential had no distinct effect on the foam stability, but was related to the initial rate of particle adsorption at the air/water interface (Figure 2).

### 3.3. β-Lg Particles at the Oil/Water Interface: Interfacial Properties and Emulsion Stabilization

#### 3.3.1. Interfacial Properties

**Figure 5:**
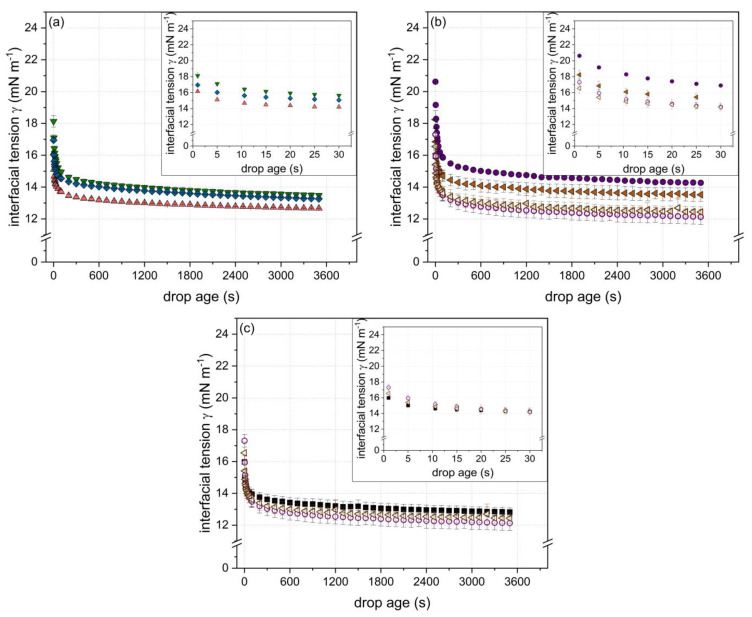
Evolution of interfacial tension (γ) in dependence of time as a function of the β-Lg particle (c = 8.2 g L^−1^) physicochemical properties: (**a**) varying degree of cross-linking (▼ 85, ▲ 92, and ◆ 109%) but similar size (~22 nm) and similar zeta potential (~−30 mV); (**b**) varying net zeta potential (● −38 and ○ −30 mV or ◀ −35 and ◁ −29 mV, respectively) but similar size (●/○ ~120 and ◀/◁ ~42 nm, respectively) and similar degree of cross-linking (~105%); (**c**) varying particle size (■ 3, ◁ 40, and ○ 119 nm) and degree of cross-linking (■ 120 and ◁/○ ~105%) but a similar zeta potential of ~−30 mV. Error bars indicate standard deviation.

Besides the air/water interface, the particles’ behavior at the oil/water interface was investigated. Figure 5a–c show the evolution of the interfacial tension as a function of the particles’ physicochemical properties (i.e., particle size, zeta potential, and degree of cross-linking (Table 2)), whereby the respective insets highlight the initial interfacial tension decrease. As can be seen, all particles reduced the oil/water interfacial tension from 24 to around 12–15 mN m^−1^.

(a) Interfacial tension as a function of degree of cross-linking

At the oil/water interface, no clear influence of the degree of covalent cross-linking (85, 92, and 109%) on the reduction in interfacial tension could be determined for particles of similar size (~22 nm) and zeta potential (~−30 mV) during a time range of 3600 s (Figure 5a). The curve corresponding to the most cross-linked particles (109%) was initially between those of lower cross-linking degree, whereas for longer adsorption times, the curves approached each other. However, a crossing over of the curves may occur at longer time scales due to the less intense reduction in interfacial tension of the more cross-linked particles (109%) after the initial phase compared to the least cross-linked ones (85%). This could indicate a limited rearrangement at the interface due to the increased number of cross-links [41,43].

(b) Interfacial tension as a function of zeta potential and particle size

As can be seen in Figure 5b, lowering the zeta potential from −38 to −30 mV or from −35 to −30 mV for particles with ~120 and ~42 nm, respectively, with similar cross-linking degrees of ~105% resulted in a significantly stronger decrease in the interfacial tension during 3600 s. This increased reduction rate can be explained by a reduction in the energy barrier of adsorption by decreasing the particles’ zeta potential [37]. Comparing the particles of different sizes but similar zeta potential (−30 mV) (non-filled symbols), no significant difference in their interfacial behavior could be detected during a measurement time of 3600 s. However, the influence of particle size in terms of diffusion might become more evident for particles of a larger magnitude of size.

Similar to the results on surface activity (Section 3.2.1), the results shown clearly demonstrate the relevance of the zeta potential in terms of the interfacial activity of β-Lg particles.

(c) Surface tension as a function of particle size and degree of cross-linking

In order to separate the influence of particle size from the effect of zeta potential, Figure 5c shows the interfacial behavior of particles of different sizes (3, 40, and 119 nm) but similar zeta potential of ~−30 mV. As can be seen, smaller-sized particles showed a faster initial reduction in the interfacial tension than larger ones, whereby the effect was only significant for the particles of 3 and 119 nm. This can be explained by the higher diffusion coefficient of the smaller particles and, thus, the faster diffusion to and adsorption at the oil/water interface. Similar results were shown in previous studies [20,44]. However, this difference disappeared for adsorption times > 20 s, as can be seen by the less intense reduction in interfacial tension of the smallest particles compared to the larger ones. In this regard, it is important to mention that the smallest particles exhibited a much higher cross-linking degree (120%) compared to the larger ones (~105%). Thus, due to the enhanced ability of the less cross-linked particles to rearrange at the oil/water interface [43], a more pronounced reduction in interfacial tension was achieved.

To conclude, the zeta potential seems to be most decisive in view of reducing the interfacial tension. Nevertheless, a decrease in particle size at a similar zeta potential resulted in a faster reduction in the initial interfacial tension and, thus, a faster adsorption at the interface. In terms of the influence of the cross-linking degree, no significant effect could be detected during the measurement time up to 3600 s.

#### 3.3.2. Emulsion Stability

Besides measuring interfacial tension, oil-in-water emulsions were prepared to evaluate the significance of specific particle properties, i.e., size, zeta potential, and degree of cross-linking. The concentration used for emulsion preparation was assumed to completely cover the oil droplet surface as an increase in β-Lg concentration (≥5 g L^−1^) did not result in a decrease in oil droplet size (results not shown). This also implies that the presence of non-adsorbed protein in the bulk phase can be assumed.

**Figure 6:**
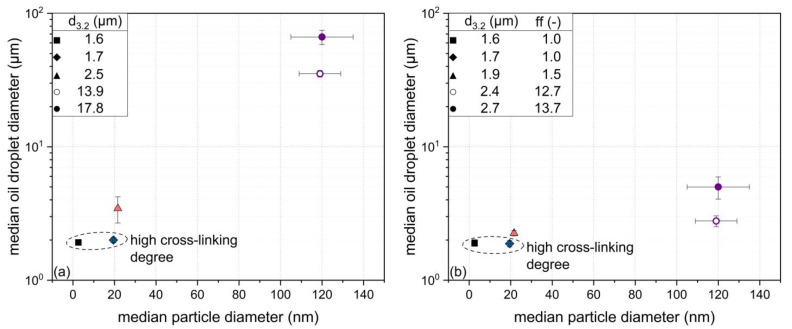
Median oil droplet diameter (and surface weighted mean (d_3.2_)) in dependence of the median particle diameter (**a**) without the addition of sodium dodecyl sulfate (SDS) and (**b**) with the addition of SDS and including the flocculation factor (*ff*) using a particle concentration of 8.2 g L^−1^ (degree of disulfide cross-linking/zeta potential: ▲ 92%/−29 mV, ○ 104%/−30 mV, ● 104%/−38 mV, ◆ 109%/−31 mV, and ■ 120%/−30 mV). Error bars indicate standard deviation.

In general, an increase in particle size (3, 22, and 120 nm) with the zeta potential set to ~−30 mV led to an increase in the oil droplet size (Figure 6a). Similar results were reported by others (e.g., [21,22]). However, differences in oil droplet size stabilized by particles of similar size (~22 or ~120 nm) could also be determined. Thus, further particle properties (i.e., zeta potential and structural flexibility) might influence the oil droplet size. Thereby, decreasing the particles’ (~120 nm) zeta potential from −38 to −30 mV resulted in the formation of smaller oil droplets (Figure 6a). Unlike expected, lower cross-linking degrees of particles of similar size (~22 nm) and zeta potential (~−30 mV) from 109 to 92% resulted in higher oil droplet sizes, whereby particles of high cross-linking degrees (≥109%) and different sizes (3 and ~22 nm) resulted in the formation of oil droplets of similar size.

As the degree of particle cross-linking affects particle–particle interactions [21], it was assumed that this could also have an impact on oil droplet aggregation. Therefore, the flocculation factor *(ff)* was calculated according to Equation (5). This allowed us to determine the degree of oil droplet aggregation as well as the actual oil droplet size.

In general, the flocculation factor increases with the tendency of oil droplets to aggregate [45]. In this regard, sodium dodecyl sulfate (SDS) was used to disaggregate flocculated oil droplets by increasing the electrostatic repulsion between oil droplets. As can be seen in Figure 6b, the addition of SDS resulted in a significant reduction in the oil droplet size in emulsions stabilized by particles of lower cross-linking degree (<104%). Furthermore, the effect of SDS was more pronounced for the larger protein particles (~120 nm). By contrast, rather rigid particles (cross-linking degree > 104%) were not affected by the addition of SDS independent of their size (i.e., 3 and ~22 nm).

An influence of the viscosity of the continuous phase on emulsion stability (i.e., creaming rate) can be excluded as no significant differences in terms of dynamic viscosity of the samples were detected. However, the viscosity may become important by using particles much larger than reported in this study as well as by using higher oil contents [22,46].

### 3.4. Comparison of the Properties of β-Lg Particles at the Air/Water and the Oil/Water Interfaces

**Figure 7:**
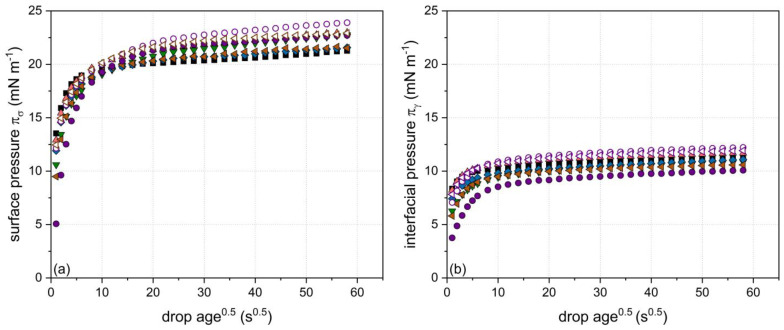
Evolution of (**a**) surface pressure π_σ_ at the air/water interface and (**b**) interfacial pressure π_γ_ at the oil/water interface as a function of the particle properties using a particle concentration of 8.2 g L^−1^ (median particle size: ■ 3, ◆ 20, ▼ 22, ▲ 22, ◁ 40, ◀ 44, ○ 119, and ● 120 nm). For further interpretation of the symbols, the reader is referred to Table 2.

In order to compare the interfacial properties of the specific particles at the air/water and oil/water interface, the surface pressure (π_σ_(t)) and the interfacial pressure (π_γ_(t)) were calculated according to Equations (3) and (4). The respective results are shown exemplarily in Figure 7a,b. As can be seen, surface pressure was found to increase faster and to higher values compared to interfacial pressure in the present set of experiments. This could be related to a lower adsorption barrier and higher packing density of particles at the air/water interface. However, contrary results were found in the literature [47,48]. In this regard, it is important to mention that the interfacial rearrangement at the oil/water interface is additionally influenced by the type of oil used [49].

In order to highlight the influence of the particle properties in the initial (t = 5 s) and final (t = 3550 s) measurement time regime, the surface pressure (π_σ_(t)) and the interfacial pressure (π_γ_(t)) values after 5 and 3550 s are included in Table 2 next to the particles’ physicochemical properties. Thereby, a decrease in zeta potential from −35 and −38 mV, respectively, to ~−30 mV for particles of ~42 and ~120 nm was observed to be decisive in terms of fast adsorption at both types of interface. By contrast, an increase in particle size up to ~120 nm at a similar zeta potential of~−30 mV was less important for both systems. A decreasing cross-linking degree was found to differently impact the surface and interfacial pressure. Whereas a decrease in the cross-linking degree increased the surface pressure at longer time scales (t = 3550 s), no significant effect on the interfacial pressure could be detected over the whole measurement duration of 3600 s. This could be an indication that particle rearrangement at oil/water interfaces requires a longer time than at the air/water interface.

Regarding the particles’ physicochemical properties being relevant for foam and emulsion stabilization, an increase in particle size as well as a decrease in cross-linking degree were found to lead to an increased foam bubble stability (Figure 3), whereas emulsion stability decreased (Figure 6). In order to explain this difference, it is important to note that the produced foams and emulsions differed in their characteristics. Emulsification was performed by high pressure homogenization, wherefore oil droplet stabilization was based on convective transport rather than diffusion. This high energy input combined with an oil-to-protein ratio close to 10:1 allowed for achieving relatively small oil droplet sizes (<5 μm) [46,50]. Compared to that, foams were produced by air sparging. Thereby, air bubbles are formed and detached from the porous frit mainly based on the equilibrium of surface tension force and buoyancy force [51]. Due to the inherent structure of the porous frit, instantaneous bubble coalescence cannot be avoided. Additionally, with the time of sparging, a foam column is formed, which is very prone to gravitational drainage, also promoting bubble coalescence, which is the main foam destabilization mechanism. Overall, combined with an air-to-protein ratio of 90:1, this leads to the formation of comparatively large air bubbles (>100 µm).

Hence, for the foams, it could be assumed that the high stability observed for large particles with low cross-linking degree, on the one hand, was due to the formation of cohesive surface films based on particle–particle interactions (adsorbed particles) [8,16]. On the other hand, non-adsorbed particles might have contributed to foam stability by means of the blocking of lamellae leading to a reduction in drainage and, therewith, less coalescence [15].

For emulsions, however, it was supposed that more pronounced inter-particle interactions occurring at lower cross-linking degrees triggered the formation of oil droplet aggregates. Unlike for foams, aggregation rather than coalescence is the main destabilization mechanism for emulsions. Resulting from the oil droplet aggregation, oil-enriched and oil-depleted areas were formed (data not shown). In this context, it is to mention that at a high oil volume fraction (i.e., ≥74%), oil droplet cluster formation would lead to an increased stability (e.g., increase in viscosity), whereas for foams, a high air volume fraction rather provokes an opposite effect [7,52].

## 4. Conclusions

In this study, we aimed to assess the specific role of particle size, zeta potential, and cross-linking degree of purified β-Lg particles decoupled from each other in the stabilization of air/water and oil/water interfaces. This was supposed to allow to (i) determine and evaluate the influence of individual particle properties under the exclusion of overlapping effects and (ii) to compare the behavior of standardized particles at different types of interfaces. With regard to the particles’ behavior at both the air/water and the oil/water interface, zeta potential was found to be decisive during the initial adsorption regime, i.e., a fast increase in the initial surface/interfacial pressure was linked to reduced zeta potential. Unlike expected, at constant zeta potential, particle size did not distinctly affect the initial adsorption regime. In terms of foam and emulsion stabilization, the degree of cross-linking was found to be key, whereby contradictory effects were observed for the two systems. While an increased flexibility resulted in an increased foam stability, emulsion stability was decreased due to oil droplet aggregation.

Overall, it can be concluded that a specific type of protein particle has a different impact on stabilizing foams compared to emulsions, wherefore explanatory approaches cannot be used interchangeably. Nonetheless, the systematic investigation of the stabilization of air/water and oil/water interfaces offers a particle ‘toolbox’ which opens new opportunities to precisely design particles for specific applications, e.g., the stabilization of air/water and oil/water interfaces.

## Figures and Tables

**Table 1:** Median particle size (d_50.3_) and degree of denaturation (DD) in dependence of heating pH and NaCl concentration of heat-treated (80 °C/90 min) β-Lg solutions (10 g L^−1^) (n.d.—not determined).

pH	NaCl	d_50.3_	DD
(-)	(mM)	(nm)	(%)
5.8	0	80 ± 3	88 ± 1
6.2	0	41 ± 9	82 ± 1
6.8	0	20 ± 1	81 ± 1
7.6	0	n.d.	n.d.
8.5	0	3 ± 1	69 ± 4
5.8	20	495 ± 72	96 ± 1
6.8	20	23 ± 1	96 ± 1
8.5	20	6 ± 1	83 ± 1
5.8	60	>1000	n.d.
6.2	60	189 ± 19	>96
6.8	60	28 ± 4	>96
7.6	60	28 ± 1	>96
8.5	60	25 ± 1	96 ± 1

**Table 2:** Characteristics (median particle size (d_50.3_), polydispersity index (PDI), degree of cross-linking (DC), and zeta potential (ZP)) and interfacial properties (surface (π _σ_) and interfacial pressure (π _γ_) after 5 and 3550 s) of purified (*DD* ≥ 96% and κ < 1 mS cm^−1^) β-Lg particles formed by heat treatment (80 °C/90 min) under variation of pH and NaCl.

Formation			Purified Particle Characteristics					Interfacial Properties			
pH	NaCl	Icon	pH	d_50.3_	PDI	DC	ZP	π _σ 5 s_	π _σ 3550 s_	π _γ 5 s_	π _γ 3550 s_
(-)	(mV)		(-)	(nm)	(-)	(%)	(mV)	(mN m^−1^)	(mN m^−1^)	(mN m^−1^)	(mN m^−1^)
5.8	0	●	6.8	120 ± 14	0.19 ± 0.02	104 ± 0.1	−38 ± 1.1	10.4 ± 0.3	22.8 ± 0.2	5.2 ± 0.1	10.4 ± 0.4
5.8	0	○	6.2	119 ± 10	0.19 ± 0.01	104 ± 0.1	−30 ± 0.7	15.8 ± 1.3	24.3 ± 0.5	8.4 ± 0.4	12.2 ± 0.5
6.2	0	◀	6.8	44 ± 9	0.19 ± 0.02	106 ± 4.3	−35 ± 1.7	13.7 ± 0.9	21.6 ± 0.3	7.2 ± 0.1	10.6 ± 0.1
6.2	0	◁	6.3	40 ± 7	0.19 ± 0.02	106 ± 4.3	−29 ± 0.4	15.4 ± 0.1	23.0 ± 0.4	8.8 ± 0.8	11.8 ± 0.5
6.8	0	◆	6.8	20 ± 1	0.26 ± 0.00	109 ± 1.0	−31 ± 0.2	15.0 ± 0.1	21.6 ± 0.1	8.3 ± 0.1	11.1 ± 0.1
8.5	0	■	6.8	3 ± 1	0.64 ± 0.11	120 ± 6.5	−30 ± 0.5	16.2 ± 0.2	21.4 ± 0.2	9.3 ± 0.3	11.5 ± 0.3
6.8	20	▼	6.8	22 ± 1	0.47 ± 0.02	85 ± 1.1	−29 ± 1.3	14.1 ± 0.6	22.9 ± 0.9	7.3 ± 0.2	10.9 ± 0.1
8.5	60	▲	6.8	22 ± 2	0.41 ± 0.04	92 ± 3.2	−29 ± 2.2	15.9 ± 1.0	23.1 ± 0.6	9.3 ± 0.1	11.7 ± 0.1

## Data Availability

No data supporting material is available.
